# Doxepin and imipramine but not fluoxetine reduce the activity of the rat glutamate transporter EAAT3 expressed in *Xenopus* oocytes

**DOI:** 10.1186/s12871-015-0098-5

**Published:** 2015-08-08

**Authors:** Hye Jin Park, Hee Jung Baik, Dong Yeon Kim, Guie Yong Lee, Jae Hee Woo, Zhiyi Zuo, Rack Kyung Chung

**Affiliations:** 1 Dasom anesthesia and analgesia practice association, Seoul, Republic of Korea; 2 Department of anesthesiology and pain medicine, School of Medicine, Ewha Womans University, Seoul, Republic of Korea; 3 Department of Anesthesiology, University of Virginia Health System, Charlottesville, VA USA

## Abstract

**Background:**

Many researchers have suggested that the glutamatergic system may be involved in the effects of antidepressant therapies. We investigated the effects of doxepin, imipramine, and fluoxetine on the excitatory amino acid transporter type 3 (EAAT3).

**Methods:**

EAAT3 was expressed in *Xenopus* oocytes by injection of EAAT3 mRNA. Membrane currents were recorded after application of L-glutamate (30 μM) in the presence or absence of various concentrations of doxepin, imipramine, and fluoxetine. To study the effects of protein kinase C (PKC) activation on EAAT3 activity, oocytes were pre-incubated with phorbol 12-myristate-13-acetate (PMA) before application of imipramine and doxepin.

**Results:**

Doxepin at 0.063–1.58 μM significantly decreased EAAT3 activity. Imipramine reduced EAAT3 activity in a concentration-dependent manner at 0.16–0.95 μM. However, fluoxetine did not affect EAAT3 activity, and PMA increased EAAT3 activity. At 0.32 μM, imipramine caused an equivalent decrease in EAAT3 activity in the presence or absence of PMA. However, 0.79 μM doxepin did not abolish the enhancement of EAAT3 activity by PMA.

**Conclusions:**

We showed that doxepin and imipramine, but not fluoxetine, inhibited EAAT3 activity at clinically relevant concentrations. This reveals a novel mechanism of action for doxepin and imipramine; that they increase glutamatergic neurotransmission. PKC may be involved in the effects of doxepin on EAAT3, but is not involved in the effects of imipramine at the concentrations studied.

## Background

Glutamate is a major excitatory amino acid neurotransmitter in the central nervous system (CNS), and can cause excitotoxic neuronal damage at high extracellular concentrations. Excitatory amino acid transporters (EAATs) are present in the plasma membranes of neurons and glial cells, and take up extracellular glutamate to regulate glutamatergic transmission and prevent glutamate-mediated neuronal damage [1–3]. Five subtypes of EAATs have now been identified (EAAT1–5): EAAT1 and EAAT2 are found in glia, EAAT3 and EAAT4 are found in neurons, and EAAT5 is distributed primarily in the retina [1]. EAAT3 is the most abundant neuronal transporter, especially in the hippocampus, basal ganglia, and olfactory bulb [4, 5]. EAAT3 is also highly expressed outside the CNS, such as in the kidney, placenta, intestine, pancreas, bone, and heart.

Evidence from preclinical and clinical research suggests that glutamate plays a key role in major depressive disorder (MDD) and in antidepressant therapy [6–9]. Antidepressants are effective for treating MDD and are also used in chronic pain, including diabetic neuropathy, postherpetic neuralgia, and fibromyalgia [10, 11]. The effects of tricyclic antidepressants (TCAs) are primarily mediated by inhibition of neuronal reuptake of norepinephrine and serotonin into synaptic nerve terminals. Selective serotonin reuptake inhibitors (SSRIs), which are newer antidepressants, exert their therapeutic effects by selectively inhibiting serotonin reuptake [12]. However, several studies have also indicated the involvement of the glutamatergic system in the action of these antidepressants [13–15]. Recently, Baik *et al.* [16] suggested that amitriptyline, a TCA, inhibits EAAT3 activity, and that protein kinase C (PKC) may not be involved in this effect. However, whether other antidepressants exert the same effect on EAAT3 activity remains unclear. Although amitriptyline has similar reuptake inhibitory potencies for serotonin and norepinephrine, other antidepressants have different selectivity ratios for serotonin and norepinephrine reuptake [17]. More recent findings suggest a relationship between aminergic and glutamatergic systems [13, 18, 19]. Antidepressants with differential effects on noradrenaline and serotonin reuptake do not seem to have identical effects on function or membrane expression of glutamate ionotropic receptors, such as N-methyl-D-aspartate and α-amino-3-hydroxy-5-methyl-4-isoxazolepropionic acid receptors in the hippocampus [13, 18]. We hypothesised that different types of antidepressants would have different effects on EAAT3 activity.

Doxepin (inhibits norepinephrine reuptake more than serotonin reuptake) and imipramine (preferentially inhibits serotonin reuptake) are TCAs. Fluoxetine is an SSRI. In this study, we examined the effects of the three commonly used antidepressants, doxepin, imipramine, and fluoxetine, on EAAT3 activity using the *Xenopus* oocyte expression system. We also examined the involvement of PKC, an intracellular signalling molecule that regulates EAAT3 activity [20], on the effects of these antidepressants on EAAT3 activity.

## Methods

### Oocyte preparation and injection

The study protocol was approved by the Institutional Animal Care and Use Committee at the School of Medicine, Ewha Womans University (South Korea; Approval No. MRI 08–01, December 8, 2008). The study adhered to the recommendations from the Declarations of Helsinki and internationally accepted principles for the care and use of experimental animals.

Mature female *Xenopus laevis* frogs were purchased from Kato S Science (Chiba, Japan) and fed regular frog brittle twice weekly. All reagents, unless specified below, were obtained from Sigma (St. Louis, MO, USA).

As described before [16, 21, 22], frogs were anaesthetised in 500 mL 0.2 % 3-aminobenzoic acid ethyl ester in water until unresponsive to painful stimuli (toe pinching), after which they underwent surgery on ice. Oocytes were retrieved surgically and placed immediately in calcium-free OR-2 solution. The OR-2 solution consisted of 82.5 mM NaCl, 2 mM KCl, 1 mM MgCl_2_, 5 mM HEPES, pH 7.5. The oocytes were defolliculated with gentle shaking for approximately 2 h in calcium-free OR-2 solution including 0.1 % collagenase type Ia. The oocytes were then incubated in modified Barth’s solution at 16 °C for 1 day before injection of EAAT3 mRNA. The modified Barth’s solution consisted of 88 mM NaCl, 1 mM KCl, 2.4 mM NaHCO_3_, 0.41 mM CaCl_2_, 0.82 mM MgSO_4_, 0.3 mM Ca(NO_3_)_2_, 0.1 mM gentamicin, and 15 mM HEPES, pH 7.5.

A rat EAAT3 cDNA construct was provided by Dr. Mattias A. Hediger (Brigham and Women’s Hospital, Boston, MA, USA). The cDNA was subcloned into a commercial vector (BluescriptSKm). The plasmid DNA was linearised with a restriction enzyme (Not I) and mRNA was synthesised *in vitro* with a commercial kit (Ambion, Austin, TX, USA). The resulting mRNA was quantified spectrophotometrically and diluted in sterile water. This mRNA was used for the cytoplasmic injection of oocytes at a concentration of 40 ng/40 nL using an automated microinjector (Nanoliter 2000; World Precision Instruments, Sarasota, FL, USA). Oocytes were then incubated at 16 °C in modified Barth’s solution for 3 days before voltage-clamping experiments.

### Electrophysiological recordings

Experiments were performed at room temperature (21–23 °C). A single oocyte was placed in a recording chamber of <1 mL volume and was perfused with 5 mL Tyrode’s solution/min. The Tyrode’s solution consisted of 150 mM NaCl, 5 mM KCl, 2 mM CaCl_2_, 1 mM MgSO_4_, 10 mM dextrose, and 10 mM HEPES, pH 7.5 [16].

Clamping microelectrodes were pulled from capillary glass (single-barrel standard borosilicate glass tubing, World Precision Instruments) on a micropipette puller (Temperature-controlled pipette puller PIP5; HEKA Instruments Inc., Bellmore, New York, USA). Tips were broken at a diameter of approximately 10 μm and filled with 3 M KCl, resulting in a resistance of 1–3 MΩ. Oocytes were voltage-clamped using a two-electrode voltage clamp amplifier (OC725-A; Warner Corporation, New Haven, CT, USA) that was connected to a data acquisition and analysis system running on a personal computer. The acquisition system consists of a DAS-8A/D conversion board (Keithley-Metrabyte, Taunton, MA, USA). Analyses were performed with the pCLAMP7 software (Axon Instruments, Foster City, CA, USA). All measurements were performed at a holding potential of −70 mV. Oocytes that did not show a stable holding current less than 1 μA were excluded from the analysis. L-Glutamate was diluted in Tyrode’s solution and superfused over the oocyte for 25 s (5 mL/min). L-Glutamate-induced inward currents were sampled at 125 Hz for 1 min: 5 s of baseline, 25 s of L-glutamate application, and 30 s of washing with Tyrode’s solution [23]. The glutamate-induced peak currents were calculated to reflect the amount of glutamate transported. In this study we used 30 μM L-glutamate, unless indicated otherwise, because the pharmacokinetic parameter K_m_ of EAAT3 for L-glutamate was 27–30 μM in previous studies [21, 22].

### Administration of experimental chemicals

Doxepin and fluoxetine were dissolved in water, and imipramine was dissolved in methanol and then diluted in Tyrode’s solution to the appropriate final concentration (doxepin: 20, 50, 100, 150, 250, or 500 ng/mL, corresponding to 0.063, 0.16, 0.32, 0.47, 0.79, or 1.58 μM, respectively; imipramine: 20, 50, 100, 150, or 300 ng/mL, corresponding to 0.063, 0.16, 0.32, 0.47, or 0.95 μM, respectively; and fluoxetine: 30, 100, 150, 300, 500, or 800 ng/mL, corresponding to 0.087, 0.29, 0.43, 0.87, 1.45, or 2.31 μM, respectively), which encompassed the clinically relevant plasma concentrations for each drug [24, 25]. In control experiments, oocytes were perfused with Tyrode’s solution for 4 min before application of Tyrode’s solution containing L-glutamate for the electrophysiological recordings. In the antidepressant-treated group, oocytes were perfused with Tyrode’s solution for the first minute for stabilisation, followed by Tyrode’s solution containing antidepressant for 3 min before application of Tyrode’s solution containing L-glutamate for the electrophysiological recording. To study the effects of PKC activation on EAAT3 activity, oocytes were pre-incubated with 100 nM phorbol 12-myristate-13-acetate (PMA), a PKC activator, for 10 min before recording. Because fluoxetine did not show any significant effect on EAAT3 activity at any tested concentration, we excluded it from this study. To investigate whether there was an interaction between PMA and antidepressants, PMA-treated oocytes were exposed to 0.79 μM doxepin and 0.32 μM imipramine, as described above. These concentrations are within the therapeutic ranges of doxepin and imipramine.

### Data analysis

Each experimental condition was performed with oocytes from at least three different frogs. Because the expression level of transporter proteins in oocytes of different batches may vary, variability in responses among batches of oocytes is common. Thus, responses were normalised to the mean value of the same-day controls for each batch. Responses are reported as means. Statistical analyses were performed using SPSS version 17.0 (SPSS Inc., Chicago, IL, USA) and all data were expressed as mean ± S.E.M. Unpaired Student’s *t*-test was preformed to investigate differences in EAAT3 activity between each drug concentration and control. One-way analysis of variance followed by the Duncan test was used to investigate the interaction between PMA and antidepressants. Differences were considered significant at *p* < 0.05. Dose–response curves were plotted, and IC_50_ was calculated using GraphPad Prism (ver. 5.0; GraphPad Software, San Diego, CA, USA).

## Results

**Fig. 1:**
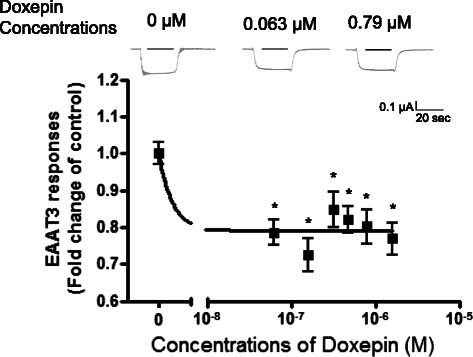
Dose–response of doxepin inhibition of excitatory amino acid transporter type 3 (EAAT3) responses to L-glutamate (30 μM). Data are means ± S.E.M. (*n* = 25 or 26 for each data point). *p < 0.05 *vs.* control

**Fig. 2:**
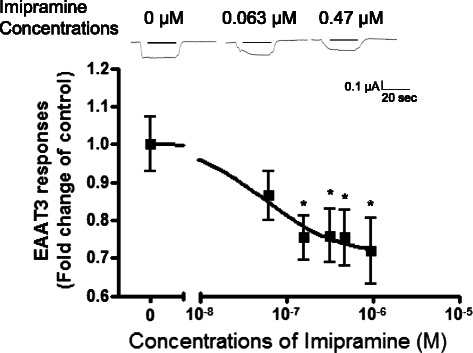
Dose–response of imipramine inhibition of EAAT3 responses to L-glutamate (30 μM). Data are means ± S.E.M. (*n* = 17–24 for each data point). *p < 0.05 *vs.* control

**Fig. 3:**
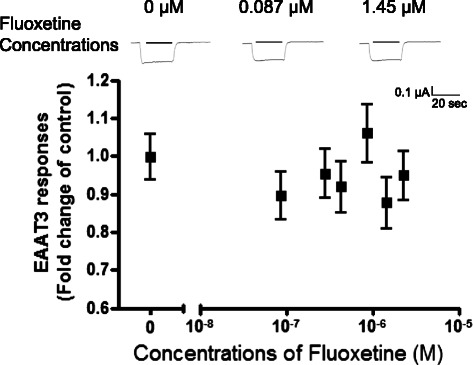
Dose–response of fluoxetine on EAAT3 responses to L-glutamate (30 μM). Data are means ± S.E.M. (*n* = 17–26 for each data point). *p < 0.05 *vs.* control

Non-injected oocytes were unresponsive to L-glutamate (data not shown), but oocytes injected with EAAT3 mRNA showed inward currents after L-glutamate was applied (Figs. 1, 2 and 3). This current was shown to be mediated *via* EAAT3 in previous studies [21, 22]. In the vehicle control experiment, 0.03 % (v/v) methanol (the highest concentration in Tyrode’s solution containing imipramine) had no effect on the current response to glutamate (0.96 ± 0.34-fold) compared with the control (1.00 ± 0.35-fold) (*n* = 10, p > 0.05).

While antidepressants alone did not induce any current in oocytes injected with or without EAAT3 mRNA (data not shown), doxepin and imipramine reduced EAAT3 responses to L-glutamate (Figs. 1 and 2). This inhibition was statistically significant at 0.063–1.58 μM doxepin (Fig. 1) (*p* = 0.000–0.008) and 0.16–0.95 μM imipramine (Fig. 2) (*p* = 0.013–0.025). The IC_50_ value (concentration required for 50 % inhibition) of imipramine was 0.056 μM (Fig. 2). However, fluoxetine showed no significant effect on EAAT3 activity at any concentration tested (Fig. 3).

**Fig. 4:**
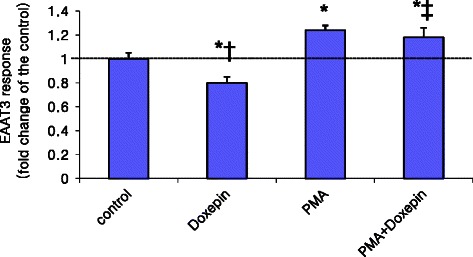
Effects of protein kinase C activation and doxepin on EAAT3 activity. EAAT3 activity was observed in the presence or absence of 0.79 μM doxepin. Data are means ± S.E.M., *n* = 25–29 in each group. *p < 0.05 *vs.* control, ^†^*p* < 0.05 *vs.* phorbol 12-myristate-13-acetate (PMA) alone, ^‡^*p* < 0.05, *vs.* doxepin alone

**Fig. 5:**
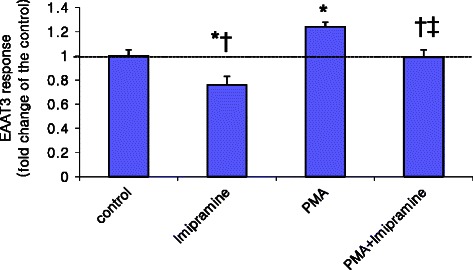
Effects of protein kinase C activation and imipramine on EAAT3 activity. EAAT3 activity was observed in the presence or absence of 0.32 μM imipramine. Data are means ± S.E.M., *n* = 19–29 in each group. *p < 0.05 *vs.* control, ^†^*p* < 0.05 *vs.* PMA alone, ^‡^*p* < 0.05 *vs.* imipramine alone

Pre-incubation of oocytes with 100 nM PMA significantly increased EAAT3 activity compared with control (1.24 ± 0.04 *vs.* 1.00 ± 0.05, *p* = 0.017, *n* = 28–29 in each group) (Figs. 4 and 5). Doxepin at 0.79 μM did not affect the EAAT3 responses to PMA (1.18 ± 0.08 in the PMA + doxepin group, 1.24 ± 0.04 in the PMA group, p > 0.05, *n* = 28 in each group) (Fig. 4). However, 0.32 μM imipramine caused an equivalent decrease in EAAT3 responses in the presence of PMA (0.99 ± 0.06 in the PMA + imipramine group, 1.24 ± 0.04 in the PMA group, *p* = 0.007, *n* = 28 or 29 in each group; Fig. 5).

## Discussion

The main finding of this study is that doxepin and imipramine, but not fluoxetine, inhibited the rat glutamate transporter EAAT3 activity at clinically relevant concentrations, and the degree of inhibition of EAAT3 activity caused by 0.32 μM imipramine was not affected by PMA. However, 0.79 μM doxepin did not abolish EAAT3 activation *via* PMA in this study.

In recent studies, glutamate seems to also be involved in the pathophysiology of MDD and in antidepressant-mediated therapeutic effects [7–9]. There is some evidence that the hippocampus may play a role in the pathophysiology of MDD. Stockmeier *et al.* [26] described a reduction in both the average soma size of pyramidal neurons and neuropil, which may contribute to volume reduction in the hippocampus of patients with MDD. Since sodium-dependent EAATs can regulate extracellular glutamate concentrations and glutamatergic neurotransmission in the CNS, EAATs may be a target for antidepressants [1]. In particular, the neuronal transporter EAAT3 has many functions, such as the regulation of inhibitory and excitatory neurotransmission, synaptic plasticity, and neuroprotection in the brain [27–29], and it accounts for about 40 % of glutamate uptake in the hippocampus [30]. Levenson *et al.* [31] showed that long-term potentiation and contextual fear conditioning in the CA1 area of the hippocampus is associated with an increase in glutamate uptake, for which an increase in plasma membrane expression of EAAT3 may be responsible.

The measurements of glutamate-induced currents have been widely used to assess the quantity of EAAT activity in the literature [16, 21, 22]. It has been shown that amitriptyline, a TCA, regulates EAAT3 mRNA expression in rat brain [32] and dose-dependently inhibits the activity of the rat glutamate transporter EAAT3 expressed in *Xenopus* oocytes [16]. However, it has not been tested whether or not other antidepressants show the same effect on EAAT3 activity. Therefore, we investigated the effects of doxepin, imipramine, and fluoxetine on EAAT3 activity.

The therapeutic ranges of plasma concentrations of doxepin and imipramine for depression are 20–250 ng/mL (0.063–0.79 μM) and 45–150 ng/mL (0.14–0.47 μM), respectively [24, 25]. Doxepin and imipramine reduced EAAT3 activity at concentrations higher than 0.063 μM for doxepin and 0.16 μM for imipramine. Thus, doxepin and imipramine can inhibit EAAT3 activity not only at clinically relevant concentrations, but also at concentrations higher than the therapeutic ranges. These results suggest that direct inhibition of EAAT3 activity may represent another plausible mechanism for doxepin and imipramine to increase glutamate concentrations in the synaptic cleft. Because reduced glutamate concentrations within the anterior cingulate cortex were seen in patients with major depressive episodes [8], inhibition of EAAT3 activity by doxepin and imipramine may contribute to their antidepressant effects. The EAAT3 responses to L-glutamate in the presence of 0.063–1.58 μM doxepin and 0.16–0.95 μM imipramine were reduced by 15–27 % and 24–28 %, respectively. These results are similar to the magnitude of decrease (20 %) by amitriptyline [16]. Because EAAT3 has been shown to be highly expressed in the kidney, placenta, intestine, pancreas, bone, and heart [1], the evidence provided by the previous study [33] that imipramine inhibits the Na^+^-dependent transport of L-glutamic acid in rat intestinal brush-border membrane is consistent with decreased EAAT3 activity caused by imipramine in this study. In a postmortem study [34], the authors detected decreased EAAT3 and EAAT4 transcripts expressions in mood disorders, which is possibly secondary to a medication effect. This report also partly supports our results.

We failed to demonstrate any significant effect of fluoxetine on EAAT3 activity, suggesting that EAAT3 may not be a target of fluoxetine, a prominent member of the SSRI class. It was demonstrated that neither acute nor chronic fluoxetine treatment in rats produces any effect on glutamate levels, but does affect brain GABA levels indirectly [35]. Their data may indicate that fluoxetine affects GABAergic transmission nonspecifically, but does not affect glutamate-related neurotransmission, which seems to support the results of the present study. Ferrero *et al.* [36] reported that chronic treatment with fluoxetine exerts a proconvulsant action in normal rats and an increase in hippocampal glutamate release. Uezato *et al.* [37] found decreased expression of the vesicular glutamate transporters in mood disorders in the entorhinal cortex and in the middle temporal gyrus. Tordera *et al.* [38] showed that a course of fluoxetine treatment for several weeks increases expression of the vesicular glutamate transporter-1, a key gene involved in the regulation of glutamate release. Thus, fluoxetine may act on the glutamatergic system by increasing glutamate release, rather than by inhibiting EAAT3 activity. Although the precise reason for these differences between TCA and SSRI is unknown, it may be attributable to the different mechanisms of action for these two classes of drugs [17].

We also studied the mechanism by which doxepin and imipramine inhibit EAAT3 activity. PKC is an intracellular signalling molecule that has been implicated in the regulation of EAAT3 activity, and several studies have shown that PKC activation increases EAAT3 activity [16, 21, 22]. The increased EAAT3 activity by PMA in this study was comparable to that shown in previous reports. One of our results, namely that the degree of inhibition of EAAT3 activity caused by 0.32 μM imipramine is not affected by PMA, suggests that 0.32 μM imipramine may not affect EAAT3 activity *via* PKC. Consistent with this, Baik *et al.* [16] reported that amitriptyline-induced reduction of EAAT3 activity is not mediated by PKC. However, 0.79 μM doxepin did not abolish EAAT3 activation *via* PMA in this study. These findings suggest that 0.79 μM doxepin may act on EAAT3 *via* PKC signalling. Consistent with this study, PKC may play a role in regulating EAAT3 activity [21, 22].

The possible limitations of this study include the fact that it is an *in vitro* experiment, and that we used rat mRNA instead of human mRNA to induce EAAT3 protein expression. Also, we did not measure glutamate reuptake directly. In addition, further studies are needed using PKC inhibitors to assess the effects of doxepin on EAAT3.

## Conclusions

Our findings demonstrate that doxepin and imipramine inhibit EAAT3 activity at clinically relevant concentrations. The inhibition by doxepin appears to be PKC-dependent, but the effect of imipramine on EAAT3 was not mediated *via* PKC, at least at the concentrations studied. These data identify a novel mechanism of antidepressant action for doxepin and imipramine. We also showed that fluoxetine, an SSRI, did not affect EAAT3 activity. Thus, EAAT3 may not be targeted by fluoxetine.
